# Pathophysiology for the Pediatric Critical Care Fellow: Three Representative Simulation Cases

**DOI:** 10.15766/mep_2374-8265.10931

**Published:** 2020-07-27

**Authors:** Candace Mannarino, Erin Bradley, Amanda Puro, Deborah Sung, Katie Wolfe

**Affiliations:** 1 Fellow, Pediatric Cardiac Critical Care Medicine, Northwestern University Feinberg School of Medicine; Fellow, Pediatric Cardiac Critical Care Medicine, Ann & Robert H. Lurie Children's Hospital of Chicago; 2 Clinical Instructor, Pediatric Critical Care Medicine, Neonatology Associates of Atlanta PC; Clinical Instructor, Pediatric Critical Care Medicine, Children's Healthcare of Atlanta; 3 Instructor, Pediatric Critical Care Medicine, Dell Children's Medical Center of Central Texas; 4 Fellow, Pediatric Critical Care Medicine, Northwestern University Feinberg School of Medicine; Fellow, Pediatric Cardiac Critical Care Medicine, Ann & Robert H. Lurie Children's Hospital of Chicago; 5 Instructor, Pediatric Critical Care Medicine, Northwestern University Feinberg School of Medicine; Instructor, Pediatric Critical Care Medicine, Ann & Robert H. Lurie Children's Hospital of Chicago

**Keywords:** Pediatric Critical Care Medicine, Simulation, Pathophysiology, Curriculum Development, Evidence-Based Medicine, Knowledge Translation

## Abstract

**Introduction:**

During the course of fellowship training, pediatric critical care fellows are expected to develop a broad and in-depth understanding of the pathophysiology of multiple disease processes. The simulation-based pediatric critical care pathophysiology curriculum we present uses scenarios created by pediatric critical care fellows to teach complex pathophysiology.

**Methods:**

Each of the three representative cases presented covered a specific pathophysiologic process and required participants to acutely manage (1) an 18-year-old patient with altered mental status in the setting of hepatic encephalopathy; (2) an 8-year-old patient with sepsis, coagulopathy, and acute kidney injury; or (3) a 12-year-old patient with status epilepticus. Each case could be conducted in a simulation suite or an acute care unit bed. We assessed learners' knowledge and attitudes at the end of these simulations with a structured debriefing session and via completion of an evaluation form. The simulations were then followed by a 30-minute interactive didactic session on the topic.

**Results:**

Each scenario had six fellow participants who completed evaluations. After completing each of the three case scenarios presented, the majority of participating pediatric critical care fellows indicated that the content was relevant and sufficiently challenging. They also indicated that these simulation scenarios would improve their clinical practice.

**Discussion:**

This fellow-developed simulation curriculum is novel, highlighting the relevance for critical care fellows' understanding of realistic clinical scenarios while promoting advanced management skills with a pathophysiology focus.

## Educational Objectives

By the end of this activity, learners will be able to:

1. Identify and treat cerebral edema as the underlying pathology in hepatic encephalopathy.
2. Demonstrate appropriate sedative use in a patient with liver failure.
3. Develop a differential diagnosis and treatment plan for a patient with coagulopathy in the setting of sepsis.
4. Demonstrate the ability to manage the airway, breathing, and circulation while developing a differential diagnosis and initiating a workup for status epilepticus.

## Introduction

Traditional teaching methods of pathophysiology include scheduled lectures and self-directed learning. High- and low-fidelity simulations used in multiple educational settings have been shown to be beneficial when compared to no intervention.^1,2^ Pediatric critical care medicine fellows must understand the underlying pathophysiology of a wide range of conditions and organ systems as well as recognizing and treating patients with an acute decompensation. Multiple studies have used simulations to prepare pediatric trainees (i.e., residents and fellows) in the event of a pediatric patient decompensation.^3–9^ To date, few studies have addressed the use of simulations as an integrative training method to supplement core pathophysiology topics or advanced management in pediatric critical care specifically for pediatric critical care fellows.^2^

The target learners for this curriculum are pediatric critical care fellows. The three simulation cases highlighted here include the pathophysiology of the disease process and focus more on the advanced management (e.g., postresuscitation care) of critically ill pediatric patients, which pediatric critical care fellows should understand upon completion of their fellowship.^10^ The expectation at the start of the simulation is to use this time to have fellows go beyond the airway, breathing, and circulation portion of their initial assessment and emphasize critical thinking. These cases also provide a unique contribution since they have been developed by trainees at our institution who have identified areas that they feel are important for their own training. We want our trainees to have the oppportunity to create scenarios that are directly applicable to the pediatric critical care environment and serve to stimulate discussion about the pathophysiologic process behind each case created. We believe this experience also gives our fellows, with mentored simulation guidance, the tools to develop simulations, create learning objectives, and teach their peers complex pathophysiology.

In addition to giving clinical context to these pathophysiologic concepts, participating fellows also participate in debriefing sessions that focus on the teamwork, leadership, and communication aspects of clinical care. These skills are essential to develop during fellowships in order to lead effective resusciation efforts. In addition to benefits for learners, we believe this type of interactive learning approach can help our trainees gain the valuable skill of implementing a simulation scenario. The low-stakes, safe learning environment also gives pediatric critical care trainees the opportunity to improve patient safety in real-life scenarios.^11,12^

## Methods

### Development

With our new pathophysiology approach, each month an assigned fellow developed a simulation case. The fellows were provided with the American Board of Pediatrics (ABP) subboard content outline for critical care medicine, as well as a list of cases covered in the previous year when selecting their topic. Over the course of a 3-year fellowship, fellows had the opportunity to create two to three unique simulations.

The fellow was expected to design a simulation scenario using our simulation template, which included prompts for the data needed to carry out the simulation scenario. The fellows worked with simulation facilitators (i.e., pediatric critical care faculty members with expertise in simulation) several days ahead of time for feedback and case preparation. The template case was also sent to the staff in our simulation center (KidSTAR) to help execute the simulation.

In total, this 60-minute activity included the 30-minute simulation experience and 30 minutes of didactics and discussion of the underlying pathophysiology of the simulated case. Although these simulations were meant to take place in our simulation center, they could also be performed in an emergency room, inpatient floor, or an acute-care bed depending on availability. Table 1 shows a list of the simulation scenarios, including the three illustrative cases that were developed by our pediatric critical care fellows in the first year of this pathophysiology curriculum. The topics and physiology were varied, as were the expected interventions, representing the breadth of content covered in this series. The majority of simulations were developed from real patient scenarios that the facilitating fellows found challenging, particularly interesting, or representative of important pathophysiology.

**Table 1.**
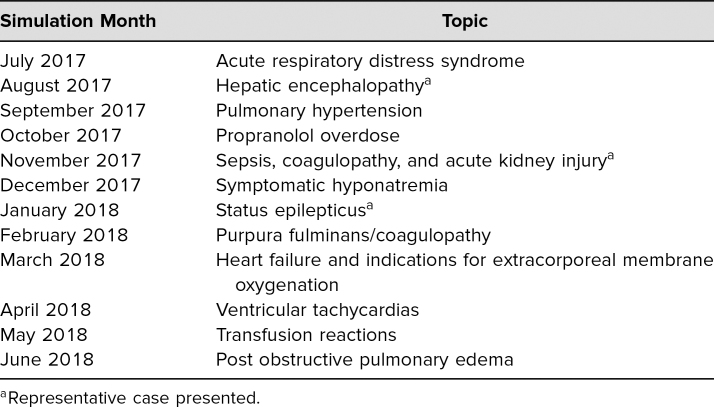
Simulation Pathophysiology Topics, July 2017-June 2018

The first representative case (Appendix A) was developed by a third-year fellow. The case was an 18-year-old male admitted to the pediatric intensive care unit (PICU) from an outside hospital emergency room with nausea, abdominal pain, and altered mental status. The expected initial management included fluid resuscitation, correction of electrolyte derangements, and sedation for agitation while simultaneously initiating a workup for the presenting symptoms. About 5–10 minutes into the case scenario, if acetaminophen toxicity has not been discussed as a potential etiology, the team was notified that a friend had disclosed that the patient had ingested a large dose of acetaminophen. The learners were encouraged to develop a differential diagnosis when they were presented with laboratory data concerning liver failure including acetaminophen toxicity, to identify sedative medications that would be appropriate or inappropriate in the setting of liver failure, and to recognize and treat cerebral edema as a potential underlying pathology in hepatic encephalopathy.

The second representative case (Appendix B) was developed by a second-year fellow. The case was an 8-year-old male who presented with fever, tachycardia, and hypotension, admitted overnight to the PICU on a norepinephrine drip. When the case started, the facilitator, acting as a nurse in the scenario, informed the learners that the patient had had an acute change of mental status and shared labs consistent with coagulopathy and acute kidney injury. The learners were expected to perform a clinical assessment and form a differential diagnosis for coagulopathy in the setting of sepsis. Learners were then guided towards advanced management, which included indications for intubation, how to manage coagulopathy, and how to utilize resources to implement their plan expeditiously and safely.

The third representative case (Appendix C) was developed by a first-year fellow. The case was a 12-year-old male with a 1-week history of muscle aches, abdominal cramping, headaches, increasing lethargy and subjective fevers for which he was admitted to the pediatric inpatient service. The scenario began with a call for a rapid response team to come to his bedside for seizure activity and concern for status epilepticus. The learners were expected to assess the patient's airway, breathing, and circulation while developing a differential diagnosis of persistently altered mental status and status epilepticus.

### Equipment/Environment

• One low- or high-fidelity mannequin (depending on the case).
• Stretcher.
• Blanket.
• Towel for shoulder roll if needed.
• Monitor operated by simulation technician displaying heart rate and arrhythmias (including ventricular tachycardia, superventricular tachycardia, sinus tachycardia, sinus bradycardia, and normal sinus rhythm), respiratory rate, oxygen saturation, and blood pressure.
• Portable defibrillator.
• Code cart with basic resuscitation equipment, including:
  ○ Code book.
  ○ Epinephrine syringe.
  ○ Sodium bicarbonate vials.
  ○ Normal saline and lactated ringer bags.
  ○ Intravenous catheters for line placement.
  ○ Non-rebreather mask.
  ○ Self-inflating bag valve mask and flow-inflating bags with mask.
  ○ Mask appropriate for mannequin size.
  ○ Mac and Miller blades for intubation.
  ○ Endotracheal tubes appropriate for mannequin size.
  ○ End tidal CO2 monitor.

### Personnel

• Simulation technician.
• At least one faculty member to assist the simulation technician and act as nurse if needed.
• One fellow running the case who can serve as nurse in the room as needed.
• Learners can divide amongst themselves if another nurse or other health professional is needed.

### Implementation

At the beginning of every simulation session, the faculty facilitator(s) reviewed the ground rules with the fellows/learners who were in attendance. Participating learners did not need extensive preparation prior to completing these sessions. The fellow running the simulation introduced the case by reading the patient history from the simulation outline. The learners were then directed into the simulation center to begin. The learners were expected to self-select their roles for the case (Appendices A–C), with one of the learners acting as the code team leader. No extra equipment was needed aside from what was listed in the case scenarios.

The facilitating fellow then conducted the pathophysiology-focused simulation serving as the nurse in the room. The faculty facilitator either assisted with running the simulation from a technology standpoint or acted as a nurse, depending on the needs of the case. The flow of the simulation included a preparatory summary statement, the simulation itself, and time for debriefing (e.g., 2–5 minutes for ground rules and case introduction, 15 minutes of simulation, and 10 minutes for debriefing). Additional learning material was discussed during the case as needed based on the given scenario. Clinical changes, laboratory data, and other useful clinical information were provided throughout the scenario by the facilitating fellow. Any vital sign changes during the scenario were visualized by changes on the simulation monitor if it was in the simulation lab environment or verbally indicated by the facilitating fellow.

### Assessment

Participants were assessed through direct observation by faculty and fellow facilitators. At the end of the simulation, the participants were required to fill out an anonymous evaluation form (Appendix D). The evaluation form focused on whether this simulation format was relevant to clinical practice, how the simulation scenario has helped the clinical practice, and the effectiveness of the debriefing session. The evaluations were scored on a 5-point Likert scale (1 = *strongly disagree*, 5 = *strongly agree*). The evaluation form also asked learners to identify their year of training (i.e., first-, second-, or third-year fellow).

### Debriefing

Debriefing was structured based on the Promoting Excellence and Reflective Learning in Simulation (PEARLS) debriefing script developed by Eppich and colleagues.^13,14^ Together, the fellow and faculty facilitators first asked the group for initial thoughts and reactions using open-ended questions and guiding all of the participants to discuss what went well and what did not go well in the case. The fellow leading the simulation reviewed key learning points. The facilitators then concluded the debriefing with final thoughts and knowledge learned from the simulation. The learners were instructed to save in-depth discussions of the medical management for the didactic portion of conference. Content specifications from the ABP content outline along with summary learning points from the didactic portion of the conference are included in the appendices.

## Results

The results for all three representative simulation cases are summarized in Table 2. The facilitators of these three cases were faculty members with an interest in medical education and simulation. The learners who participated in all three cases were pediatric critical care fellows at our training program. Each case was used only once within the 2018–2019 academic year. Fellow participants varied depending on clinical responsibilities or other time restrictions.

**Table 2.**
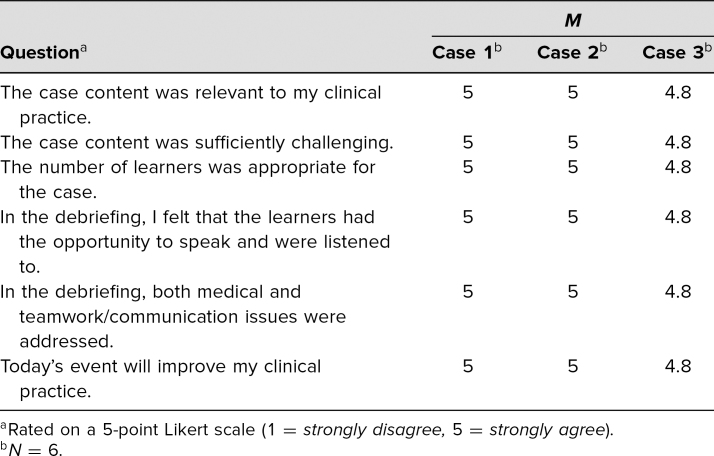
Case Rater Evaluations

For representative case 1 (Appendix A), six fellow learners (two first-years, two second-years, and two third-years) participated as nurses. Takeaways from this simulation included what sedation medications to use for a patient with liver failure, acute management of hepatic encephalopathy, and broadening the differential diagnosis for hepatic encephalopathy.

For representative case 2 (Appendix B), six fellow learners (two first-years, three second-years, and one third-year) participated as nurses. Takeaways from this simulation included that the simulation helped learners manage priorities when multiple things needed to happen simultaneously for a patient with sepsis and coagulopathy with acute kidney injury.

For representative case 3 (Appendix C), six fellow learners (three first-years, two second-years, and one third-year) participated as nurses. Takeaways from this simulation included a reminder to assign a code leader early and to remember to clearly define roles for each team member.

For cases 1 and 2, all six fellow learners indicated that they strongly agreed that the content was relevant to their clinical practice, that the case was challenging, and that this simulation event would improve their clinical practice. Additionally, all six strongly agreed that the debriefing allowed all learners the opportunity to speak, and addressed teamwork and communication issues. For case 3, five of the six fellow learners strongly agreed that the content was relevant, the case was challenging, the simulation would improve their clinical practice, and the debriefing allowed all learners the opportunity to speak, as well as addressed teamwork and communication issues.

## Discussion

An understanding of the complex pathophysiology of different disease processes is essential for pediatric critical care fellows. The representative clinical scenarios (i.e., hepatic encephalopathy, sepsis with coagulopathy and acute kidney injury, and management of status epilepticus) developed by three different pediatric critical care fellows target high-yield critical concepts that pediatric critical care clinicians will be expected to manage by the end of their training. Overall, the fellows surveyed about these case examples felt that the cases written by their cofellows were relevant to the clinical work they did every day and were sufficiently challenging. Additionally, the fellows who participated in the three cases felt the scenarios would improve their clinical practice. Therefore, these cases developed by the fellows at our institution are feasible in supplementing knowledge in a pediatric critical care pathophysiology curriculum.

Our new pathophysiology curriculum highlights the significance for critical care fellows' understanding of clinical and realistic scenarios while promoting advanced management skills with a pathophysiology focus. All three scenarios are based on experience and self-identified knowledge gaps along with the ABP content recommendations for pediatric critical care. Within 12 months of implementing this new curriculum, 12 different fellows have developed and implemented their simulations.

Limitations include ensuring an adequate number of participants while maintaining other duties if a fellow is on clinical service. The total number of participating fellows limits the applicability and power of the data shared. Future iterations of these simulations should be performed multiple times to engage all 13 members of our fellowship program. An additional limitation is that the perceptions and evaluations were self-reported, which may have introduced a degree of bias. Due to the evaluations being anonymous, we could not determine the number of cases each fellow participated in. For example, some fellows may have participated in more than one case, while others may not have participated in any. The evaluations were not specific to these cases or to this specific curriculum and therefore did not directly address the learning objectives outlined for our curriculum. Additionally, while teamwork, leadership, and communication were discussed in debriefing sessions, they were not evaluated formally, and therefore, the curriculum's impact on these skills is unknown. Future evaluations of these and other simulations should include more quantitative and qualitative data regarding the knowledge acquired and skills practiced. Long-term data about impact on clinical practice and pediatric critical care boards passing rates would also be useful but are not currently available. Finally, because these simulations were developed based on our fellows' perceived knowledge gaps, they may not be fully generalizable to different institutions and training programs.

We have learned several lessons since the implementation of our pathophysiology simulations. In order to ensure fellows were able to attend the simulation scenario, we encouraged a faculty culture of prioritizing fellows' education, especially those fellows on service. The faculty were reminded before the simulation session to let their fellows attend the conference. Our fellows overall preferred the simulation to supplement their learning both as leaders and as participants. The facilitating fellow gained a deeper understanding of the topic, often because the cases were based on real patients, and the participating fellows gained more exposure to critical management situations to guide their future management of these conditions.

Overall, fellows found the material presented clinically relevant to their practice. This experience also gave fellows an opportunity to actively engage with the faculty to try to make the experience and cases as authentic as possible. We have continued to create and implement monthly simulation sessions, creating a library of simulations that fellows can access and utilize for future learning and teaching. These simulations are mapped monthly to the ABP subboard content outline for critical care medicine for our fellows to review. The concepts are then reviewed with board-style questions at the end of the month. We will enhance our evaluation of these sessions to allow for more robust feedback and assessment of their utility for the learners.

Simulation is a feasible method by which to engage learners and teach pathophysiology during pediatric critical care training. Although many other specialties are embracing simulations as a way to supplement their curriculum, the methodology to assess performance in simulations still needs to be further explored.^2,15,16^ Further studies are needed to assess the competency of pediatric critical care fellows with the incorporation of simulations and its effect on patient safety. At this time, our data demonstrate the fellows are engaged in the learning process as they participate in simulated scenarios to teach complex pathophysiology. As an added benefit, simulation case development and implementation constitute a useful skill for fellows to acquire during training.^5^

## Appendices

Simulation Case - Hepatic Encephalopathy.docxSimulation Case - Sepsis, Coagulopathy, AKI.docxSimulation Case - Status Epilepticus.docxEvaluation Form.docx
All appendices are peer reviewed as integral parts of the Original Publication.
