# Supplementary material for: Pathophysiology for the Pediatric Critical Care Fellow: Three Representative Simulation Cases
Source: MedEdPORTAL. 2020 Jul 27;16:10931. doi: 10.15766/mep_2374-8265.10931 (PMC7384746; doi:10.15766/mep_2374-8265.10931)
Supplement: Supplementary file 1 — Simulation Case - Hepatic Encephalopathy.docxSimulation Case - Sepsis, Coagulopathy, AKI.docxSimulation Case - Status Epilepticus.docxEvaluation Form.docx [file mep_2374-8265.10931-s001.zip › A. Simulation Case - Hepatic Encephalopathy.docx]

| **Appendix A: MedEdPORTAL Simulation Case Template**  **SIMULATION CASE TITLE: Hepatic Encephalopathy**  **AUTHORS: Erin Bradley, MD**  **Katie Wolfe, MD**  **LEARNER AUDIENCE: Pediatric trainees in acute care settings** | |
| --- | --- |
| **PATIENT NAME: Richie**  **PATIENT AGE: 18-years-old**  **CHIEF COMPLAINT: Altered Mental Status**  **PHYSICAL SETTING: Pediatric Intensive Care Unit** | |
|  | |
| **Brief narrative description of case** | An 18-year-old male is admitted to the PICU from an outside hospital with a 1-day history of nausea, abdominal pain, and altered mental status. 3 weeks ago, he had fever, cough, sore throat, and myalgia. Laboratory data reveals the patient has acute liver failure.  The learners are encouraged to develop a differential diagnosis when they are presented with the laboratory data concerning for liver failure. They are expected to define sedative medications that would be appropriate or inappropriate in the setting of liver failure and recognize and treat cerebral edema as a potential underlying pathology in hepatic encephalopathy. |
| **Primary Learning Objectives** | By the end of the simulation, fellows should be able to:   1. Discuss the appropriate use of sedatives in liver failure 2. Discuss the initial management of acetaminophen ingestion 3. Identify and treat cerebral edema as the underlying pathology in hepatic encephalopathy |
| **Critical Actions** | *Initial Management*   - Perform a primary and secondary survey - Stabilize the patient by establishing access and assessing fluid status for fluid resuscitation - Identify and discuss the differential for altered mental status - Send initial labs including urine drug screen, acetaminophen level, liver function, and coagulation studies - Discuss medications for sedation (while monitoring hemodynamics)   *Advanced Management*   - Prepare for intubation |
| **Learner Preparation or Prework** | General knowledge of PALS  General knowledge of the management of liver failure  General knowledge of different sedation agents to use for intubation |

| Initial Presentation | | | |
| --- | --- | --- | --- |
| **Initial vital signs** | *Rhythm* Sinus tachycardia  *HR* 120 bpm  *BP* 95/55  *O2 Sat* 100% on RA  *RR* 22/min  *Temp* 37.5 C  *Weight* 70 kg | | |
| **Overall Setting and Appearance** | Mannequin on stretcher- the participants are told that the patient is being combative and trying to bite the nurse | | |
| **Confederates (e.g., standardized participants) and their roles in the room at case start** | Doctor #1: Team Leader  Doctor #2: Airway duty  Doctor #3: Survey duty  Nurse #1: Medication Administration Nurse (may also be the medication preparation nurse and/or documenting nurse depending on the learner census)  Nurse #2: Medication Preparation Nurse  Nurse #3 Documenting Nurse  Instructor #1: Simulation and debriefing facilitator  Instructor #2: If a 2^nd^ instructor is present, he or she can act as a parent | | |
| **HPI** | Information volunteered by Instructor #1: an 18-year-old male is admitted to the PICU from an outside hospital with a 1-day history of nausea, abdominal pain, and altered mental status. 3 weeks ago, he had fever, cough, sore throat, and myalgia. Laboratory data reveals the patient has acute liver failure.  Labs obtained by outside hospital:  *Labs and imaging given if asked for specifically*  White blood cell count 11, Hemoglobin 13.4 and platelets 164  Sodium 134 / Potassium 4 Chloride 97 / Bicarbonate 21 BUN 15/ Creatinine 1.0 Glucose 81  Aspartate aminotransferase (AST) 5060  Alanine aminotransferase (ALT) 3950  GGT 194  Total bilirubin 5.3  Alkaline phosphatase 425  Albumin 2.4  INR 3/ Prothrombin time 32.6  Acetaminophen Level: 72 (estimated 18 hours post-ingestion)  Imaging: None | | |
| **Past Medical/Surgical History** | **Medications** | **Allergies** | **Family History** |
| History of depression  No hospitalizations or history of previous suicide attempts | Not currently taking any long-term medications | NKDA | History of depression on maternal side |
| **Physical Examination** | | | |
| **General** | Sleepy, combative, and tries to bite nurse when touched | | |
| **HEENT** | Pupils equal and reactive to light | | |
| **Neck** | Mild lymphadenopathy | | |
| **Lungs** | Clear to auscultation bilaterally | | |
| **Cardiovascular** | Tachycardic, no murmurs, rubs or gallops noted  No edema  +2 pulses. | | |
| **Abdomen** | Mild distention and tender to palpation | | |
| **Neurological** | Sleepy but no focal neurological deficits | | |
| **Skin** | Capillary refill 3 seconds | | |
| **GU** | No signs of trauma | | |
| **Psychiatric** | Combative and not redirectable | | |

| Instructor Notes - Changes and CASE Branch Points | | |
| --- | --- | --- |
| **Intervention / Time point** | **Change in Case** | **Additional Information** |
| 5 minutes in the case | If acetaminophen level is not requested, the confederate nurse can state that after admission to the emergency department, the friend accompanying the patient discloses acetaminophen ingestion | Acetaminophen Level: 72 (estimated 18 hours post-ingestion) indicating probable toxicity based on Rumack-Matthew Nomogram |
| 10 minutes into the case | The nurse states: “Hey, he’s not responding to me anymore.” | Patient does not respond to painful stimuli |
| If a benzodiazepine drug given for sedation | BP is 80/40 and HR 95  BP does not respond to 20ml/kg bolus of crystalloid | Nurse alerts the provider that the blood pressure is lower |
| Team member should ask for ammonia since not given | Ammonia level 120 |  |

**Ideal Scenario Flow**

The learners enter the room and are told the patient is combative. They ask for the patient to be placed on monitors and ask about the patient’s intravenous access status and assess fluid status for fluid resuscitation. The initial management should include fluid resuscitation, correction of electrolytes, and sedation for agitation while simultaneously initiating workup for the presenting symptoms. If they give a benzodiazepine for agitation, the patient has relative hypotension which the learners should respond with fluid resuscitation. They discuss the differential of his altered mental status. The learners also acknowledge the history of viral process and think about potential acetaminophen ingestion. The learners will request a basic metabolic panel, liver function test, urine toxicology screen, acetaminophen level and an ammonia level. The learners recognize inability to protect airway after nurse states that the patient is no longer responsive after prompting. The learners prepare to intubate with sedation medications. The learners are mindful to avoid benzodiazepines in acute liver failure or understand the risks of proceeding with that class of medication.

**Anticipated Management Mistakes**

1. Administration of benzodiazepines: some of the learners may want to sedate with a benzodiazepine which the patient could develop severe hypotension and decline of mental status. Benzodiazepines should be used with caution in liver failure since this class of medication can result in prolonged hemodynamic compromise or worsening alterations in mental status as hepatic metabolism is impaired.
2. Failure to request an acetaminophen level: if this level is not ordered, it could result in inappropriate therapy.
3. Failure to request ammonia level and other labs indicative of liver failure (AST, ALT, and a coagulation panel). Acute hepatic failure has a non-specific presentation and has a broad differential diagnosis (ingestion/toxicity, infection and metabolic).
4. Failure to recognize declining mental status as indication for intubation and possible hyperosmolar therapy: learners may find it helpful for either the facilitator or nurse to comment on the declining mental status and low rate of breathing. Hepatic encephalopathy is secondary to multiple mechanisms but there should high suspicion for cerebral edema in patient with liver failure and altered mental status. The initial management includes maintaining adequate cerebral perfusion pressure, utilizing hyperosmolar therapies, correcting electrolytes derangements, decreasing overall metabolic demand with fever control, sedation and/or intubation.
