# Supplementary material for: Pathophysiology for the Pediatric Critical Care Fellow: Three Representative Simulation Cases
Source: MedEdPORTAL. 2020 Jul 27;16:10931. doi: 10.15766/mep_2374-8265.10931 (PMC7384746; doi:10.15766/mep_2374-8265.10931)
Supplement: Supplementary file 1 — Simulation Case - Hepatic Encephalopathy.docxSimulation Case - Sepsis, Coagulopathy, AKI.docxSimulation Case - Status Epilepticus.docxEvaluation Form.docx [file mep_2374-8265.10931-s001.zip › B. Simulation Case - Sepsis, Coagulopathy, AKI.docx]

| Appendix B: MedEdPORTAL Simulation Case Template  SIMULATION CASE TITLE: Sepsis, Coagulopathy, and Acute Kidney Injury  AUTHORS: Amanda Puro, MD  Katie Wolfe, MD  LEARNER AUDIENCE: Pediatric trainees in acute care settings | |
| --- | --- |
| PATIENT NAME: Matthew  PATIENT AGE: 8 years old  CHIEF COMPLAINT: Fever and hypotension  PHYSICAL SETTING: Pediatric Intensive Care Unit | |
|  | |
| Brief narrative description of case | An 8-year-old previously healthy male (no known past medical history and had moved from Africa a few months ago) presents with fever, tachycardia and hypotension. He was treated in emergency room for uncompensated septic shock and was admitted overnight to the pediatric intensive care unit (PICU) on a norepinephrine drip. 12 hours after admission he has an acute change of mental status and recent labs now has a coagulopathy, and elevated BUN and creatinine. .  The learners are to perform a clinical assessment, discuss indications for intubation, develop a differential diagnosis for coagulopathy in the setting of sepsis. |
| Primary Learning Objectives | By the end of the simulation, fellows should be able to:  1. Stabilize the patient by establishing access and assessing fluid status for fluid resuscitation  2. Develop a differential diagnosis and treatment plan for a patient with coagulopathy in the setting of sepsis  3. Discuss ways to monitor of neurologic exam in intubated and sedated patient such as near-infrared spectroscopy (NIRS) and electroencephalogram (EEG), utility of brain imaging (CT versus fast MRI) |
| Critical Actions | Initial Management:   - Perform an initial primary survey: Airway, Breathing, and Circulation (ABCs) - Learners should immediately recognize that the patient is not protecting his airway and prepare to intubate - Stabilize the patient by establishing access and assessing fluid status for fluid resuscitation - Ask bedside RN for clarification and details regarding urine output, progression of mental status changes, vital sign trends, and if any other concerns   Advanced management   - Discuss ways to monitor of neurologic exam in intubated and sedated patient including NIRS and EEG. - Discuss the pathophysiology of disseminated intravascular coagulation (DIC) and thrombocytopenia-associated multiple-organ failure (TAMOF) |
| Learner Preparation or Prework | General knowledge of PALS  General knowledge of the management of sepsis and DIC  General knowledge of different sedation agents to use for intubation |

| Initial Presentation | | | |
| --- | --- | --- | --- |
| Initial vital signs | *Rhythm* Sinus tachycardia  *HR* 130 bpm  *BP* 80/40  *O2 Sat* 90% on RA  *RR* 14/min  *Temp* 39.3 C  *Weight* 25 kg | | |
| Overall Setting and Appearance | Learners will see a mannequin on stretcher bed with a confederate as the bedside nurse | | |
| Confederates (e.g., standardized participants) and their roles in the room at case start | Doctor #1: Team Leader  Doctor #2: Airway duty  Doctor #3: Survey duty  Nurse #1: Medication Administration Nurse (may also be the medication preparation nurse and/or the documenting nurse depending on the learner census)  Nurse #2: Medication Preparation Nurse  Nurse #3 Documenting Nurse  Instructor #1: Simulation and debriefing facilitator  Instructor #2: If a 2^nd^ instructor is present, can act as a parent | | |
| HPI | *Volunteered by instructor #1 prior to going into room:*    This 8-year-old male was previously healthy but had a recent several-day history of cough, sore throat, rhinorrhea, and abdominal pain. The father notes that in the past 2 days, he has had non-bloody, non-bilious emesis and thus poor enteral intake He denies diarrhea. This morning, he developed myalgias with the most pain localizing to his right knee and a fever to 103 degrees Fahrenheit which prompted parents to seek care. In the emergency department (ED) he had labs performed and received 100 milliliters/kilogram of fluid resuscitation. He was then started on a norepinephrine infusion due to persistent hypotension.  First doses of Ceftriaxone and Vancomycin have been administered.  After his initial work up in the ED, he was admitted to the PICU. This morning the patient was sleeping but able to awaken with exam and answer questions appropriately. However, when called to his bedside a few hours later, the patient was sleepier and had an acute change in mental status: he did not open his eyes, follow commands or answer questions. The bedside RN also informs you that he has been oozing from his IV sites and has not urinated in about 6 hours.  *Other information and laboratory data (given if asked for):*  Urine output is <100 mL in 12 hours  Sodium 143/Potassium 5.2  Chloride 110/Bicarbonate 19  BUN 11 / Creatinine (Cr) .55 in ED 🡪 now BUN 20 / Cr 1.65  Glucose 180  CBC:  White blood cell count 19  Hemoglobin 11.8 in ED 🡪 now 9.1 in PICU  Platelet count 150 → now 125  Schistocytes present  Aspartate aminotransferase (AST) 200  Alanine aminotransferase (ALT) 300  GGT normal  Total bilirubin 5.3  Alkaline phosphatase 425  Lactate dehydrogenase 507  Albumin 2.4  INR 2 / Prothrombin time 32.6/ activated partial thromboplastin time 70  +Rhino/enterovirus  Blood culture Gram + cocci (positive result 24 hours after admission)  Initial ED imaging evaluation:  Chest x-ray was read as a viral process but no focal consolidation. No pneumothorax (no image needed for simulation)  Abdominal ultrasound was read as negative  Right knee x-ray: no fracture  Doppler of right lower extremity for increased right leg swelling: negative | | |
| Past Medical/Surgical History | Medications | Allergies | Family History |
| No history of joint infections  No history of autoimmune diseases  Vaccines up to date | None | NKDA | No family history of autoimmune diseases |

| Physical Examination | |
| --- | --- |
| General | Somnolent but wakes up to painful stimuli |
| HEENT | Pupils equal and reactive to light, sclerae injected, extra ocular movements intact when move, but does not make eye contact, and lips dry with moist mucus membranes |
| Neck | + Meningismus (positive Kernig sign) |
| Lungs | Transmitted upper airway sounds, intermittent irregular breathing pattern, good air entry |
| Cardiovascular | Normal S1 S2 heart sounds  Tachycardic, and regular rhythm, no murmurs |
| Abdomen | Stirs with palpation in the left lower quadrant  Diffuse distention but soft  No hepatomegaly appreciated |
| Neurological | Eyes closed throughout exam  Doesn’t follow commands or answer questions  Moans, with Kernig test and abdominal exam, but speech is not coherent  Stirs minimally to sternal rub  Pupils 3mm equal and reactive to light  Tongue midline  Normal symmetric tone  Intermittently localizes to noxious stimuli in extremities, +2 patellar and brachioradialis reflexes  Toes down going bilaterally |
| Skin | Cool hands and feet with capillary refill of 4 seconds |
| GU | Normal external male genitalia |
| Musculoskeletal | Right knee decreased mobility but no warmth or erythema |

| Instructor Notes - Changes and Case Branch Points | | |
| --- | --- | --- |
| Intervention / Time point | Change in Case | Additional Information |
| 1-2 minutes into the case | If learners do not recognize that the patient is not protecting his airway | The nurse can state: “Hey, his breathing is really slow. Do you think we should intubate?” |
| If given a benzodiazepine | Patient will become hypotensive (BP 70/30) | The nurse alerts the providers that the blood pressure is lower |
| 5-10 minutes into the case, if urine output is not discussed or only briefly mentioned | The bedside RN also informs the learners that he has been oozing from his IV sites and has not urinated in about 6 hours | The nurse can ask: “What should we do about his urine output? He hasn’t peed in 6 hours” |
| After intubation | If learners do not discuss additional workup | The nurse can ask: “I have extra blood from his recent blood draw if you want me to send additional labs.” |

Ideal Scenario Flow

The learners enter the room to find the patient somnolent. They recognize that the patient has a Glasgow Coma Scale less than 8 and prepare to intubate. They recognize that they should avoid benzodiazepines since this class of medication is metabolized by the liver (he has elevated liver enzymes and a coagulopathy), and they have the potential to be cardiac depressants (he has been hypotensive requiring a vasoactive medication). The team acknowledges that there is acute kidney injury (AKI). After the patient is intubated, the learners should discuss neuroprotective strategies and methods of monitoring the neurologic exam (EEG, NIRS etc.). The team should also discuss what additional workup they would like to send in the setting of worsening coagulopathy, AKI, altered mental status, and shock including repeating coagulation studies and review of the blood smear. If there is enough time, this will lead the team to also discuss the pathophysiology of disseminated intravascular coagulation (DIC) and thrombocytopenia-associated multiple-organ failure (TAMOF).

Anticipated Management Mistakes

1. Administration of benzodiazepines: some of the learners may discuss giving a benzodiazepine which the patient could develop severe hypotension.
2. Failure to recognize oliguria: learners may fail to acknowledge that the patient is having low urine output. This can lead to develop worsening fluid overload and more difficulties with oxygenation and ventilation. The bedside nurse can assist by stating that she’s realized the patient hasn’t urinated in 6 hours and had less than 100ml urine in the last 12 hours.
3. Failure to assess and discuss risk of bleeding: learners may not discuss bleeding risk in this patient who is coagulopathic. The bedside nurse or instructor can comment on oozing or bleeding from IV sites. The management of children with Multiple Organ Dysfunction Syndrome (MODS) and DIC is complex and requires balancing risks and benefits of interventions (maintaining neuroprotection and placing invasive lines in the setting of coagulopathy). TAMOF is a spectrum of autoimmune phenomenon resulting in microvascular thrombosis, thrombocytopenia. Therapies for TAMOF include: immunomodulators and plasma exchange.
