# Supplementary material for: Pathophysiology for the Pediatric Critical Care Fellow: Three Representative Simulation Cases
Source: MedEdPORTAL. 2020 Jul 27;16:10931. doi: 10.15766/mep_2374-8265.10931 (PMC7384746; doi:10.15766/mep_2374-8265.10931)
Supplement: Supplementary file 1 — Simulation Case - Hepatic Encephalopathy.docxSimulation Case - Sepsis, Coagulopathy, AKI.docxSimulation Case - Status Epilepticus.docxEvaluation Form.docx [file mep_2374-8265.10931-s001.zip › C. Simulation Case - Status Epilepticus.docx]

| **Appendix C: MedEdPORTAL Simulation Case Template**  **SIMULATION CASE TITLE: Status Epilepticus**  **AUTHORS: Deborah Sung, MD**  **Katie Wolfe, MD**  **LEARNER AUDIENCE: Pediatric trainees in acute care settings** | |
| --- | --- |
| **PATIENT NAME: Justin**  **PATIENT AGE: 12-years-old**  **CHIEF COMPLAINT: Status Epilepticus**  **PHYSICAL SETTING: Inpatient floor** | |
|  | |
| **Brief narrative description of case** | A 12-year-old male presents with a 1-week history of muscle aches, abdominal cramping, headaches, increasing lethargy and “feeling hot to the touch.” After developing facial twitching for 5 minutes and emesis in the emergency room, he was given 0.1mg/kg of IM lorazepam for a concern for seizures. He was admitted to the pediatric inpatient service. Since being admitted, he has not returned to his neurological baseline and started seizing again.  The learners are expected to assess the patient’s airway, breathing and circulation while developing a differential diagnosis of persistently altered mental status and status epilepticus. |
| **Primary Learning Objectives** | By the end of the simulation, fellows should be able to:   1. Demonstrate the ability to manage airway, breathing and circulation while developing a differential diagnosis for seizures 2. Describe and explain the workup for seizures (labs, imaging, and cerebral spinal fluid (CSF) studies) 3. Describe appropriate use anti-convulsant medications in the setting of status epilepticus |
| **Critical Actions** | **Initial Management:**   - Perform the initial primary survey: Airway, Breathing, and Circulation (ABCs) - Discuss the status epilepticus pathway maintained at our institution (lorazepam 0.1mg/kg followed by fosphenytoin 20 PE/kg) - Stabilize the patient by establishing access and assessing fluid status for fluid resuscitation, respiratory exam for impending respiratory failure, and neurologic exam   **Advanced Management:**   - Discuss obtaining a CSF sample while considering a plan for the airway and sedation management - Intubation for respiratory failure secondary to status epilepticus |
| **Learner Preparation or Prework** | General knowledge of PALS  General knowledge of the management of status epilepticus  General knowledge of different sedation agents to use for intubation |

| Initial Presentation | | | |
| --- | --- | --- | --- |
| **Initial vital signs** | *Rhythm* Sinus tachycardia  *HR* 140 bpm  *BP* 140/98  *O2 Sat* 91% on RA  *RR* 26/min  *Temp* 37.3 C  *Weight* 30 kg | | |
| **Overall Setting and Appearance** | Learners will see a mannequin on stretcher bed with generalized tonic-clonic movements (depending on fidelity of simulator) or that they are told the patient is seizing with confederate as the bedside nurse. | | |
| **Confederates (e.g., standardized participants) and their roles in the room at case start** | Doctor #1: Team Leader  Doctor #2: Airway duty  Doctor #3: Survey duty  Nurse #1: Medication Administration Nurse (may also be the medication preparation nurse and/or the documenting nurse depending on the learner census)  Nurse #2: Medication Preparation Nurse  Nurse #3 Documenting Nurse  Instructor #1: Simulation and debriefing facilitator  Instructor #2: If a 2^nd^ instructor is present, can act as a parent | | |
| **HPI** | *Volunteered by nurse prior to going into room:*  A 12-year-old male presents with a 1-week history of muscle aches, abdominal cramping, headaches, and feeling “hot to the touch.” Today, he developed facial twitching and emesis, then became unresponsive. He was brought in by Emergency Medical Services. He received one dose of IM lorazepam at 0.1mg/kg and has not returned to neurological baseline after being admitted to the inpatient pediatric floor. A rapid response team called for reoccurrence of and ongoing seizures while on inpatient floor. The seizures were described as generalized tonic-clonic movements and left eye deviation, teeth grinding, and eye twitching.  *Labs and imaging given if asked for specifically*  Labs:  Normal electrolytes: Glucose 102 Na 142  Drug screen panel (urine and serum): negative  Imaging:  Head computed tomography (CT) was normal  *no image is needed to complete the case* | | |
| **Past Medical/Surgical History** | **Medications** | **Allergies** | **Family History** |
| No past medical history Vaccines are up to date | No medications  No anti-epileptic medications | No known drug allergies | Father and paternal uncle have history of seizures when they were young |
| **Physical Examination** | | | |
| **General** | Unresponsive to verbal or tactile stimuli. General tonic clonic movements | | |
| **HEENT** | Pupils dilated and deviated to the left | | |
| **Neck** | Natural airway. Noticeable teeth grinding | | |
| **Lungs** | Lungs clear to auscultation bilaterally | | |
| **Cardiovascular** | Tachycardic  No murmurs, rubs, or gallops  Warm and well perfused  Capillary refill <2 seconds | | |
| **Abdomen** | Soft, non-tender, and non-distended | | |
| **Neurological** | Face symmetric. Bilateral eye twitching.  Jerky movements of upper and lower extremities bilaterally | | |
| **Skin** | No rashes | | |
| **GU** | No abnormalities | | |
| **Psychiatric** | Unresponsive during exam | | |

| Instructor Notes - Changes and CASE Branch Points | | |
| --- | --- | --- |
| **Intervention / Time point** | **Change in Case** | **Additional Information** |
| If lorazepam is given for seizures | O2 saturations decrease to 90% and BP decreases to 90/40 | The nurse points out that O2 sat and blood pressure are lower |
| If airway is not supported (i.e. no oxygen given, no bag/mask ventilation) | Oxygen saturations become lower (80s) and patient develops hypopnea (RR 6) following anti-convulsant medications | The nurse states that patient doesn’t seem to be breathing well |
| If Fosphenytoin is given | Seizure activity stops |  |
|  |  |  |

**Ideal Scenario Flow**

The learners are expected to assess the patient’s airway, breathing and circulation while developing a differential diagnosis of persistently altered mental status and status epilepticus. The learners will stabilize the patient by establishing access and assessing fluid status for fluid resuscitation, respiratory exam for impending respiratory failure, and neurologic exam. They should place the patient on oxygen and support breathing if the saturations are lower or the patient has hypopnea. They should administer benzodiazepines and fosphenytoin before the seizure activity stops. They should discuss an additional workup for the patient as well as indications for intubation prior to lumbar puncture and/or imaging.

**Anticipated Management Mistakes**

1. Failure to identify declining mental status as indication for intubation: learners may not identify the need for intubation only after either the facilitator or nurse comments on the declining mental status and low rate of breathing. Consistent re-evaluation of the ABCs are important in the management of seizure and alterations in mental status.
2. Failure to identify hypotension after benzodiazepine administration. Benzodiazepines are first line therapy in management of status epilepticus, typically followed by fosphenytoin. Both of these classes of medications can cause hemodynamic compromise and respiratory depression.
