# Supplementary material for: Pathophysiology for the Pediatric Critical Care Fellow: Three Representative Simulation Cases
Source: MedEdPORTAL. 2020 Jul 27;16:10931. doi: 10.15766/mep_2374-8265.10931 (PMC7384746; doi:10.15766/mep_2374-8265.10931)
Supplement: Supplementary file 1 — Simulation Case - Hepatic Encephalopathy.docxSimulation Case - Sepsis, Coagulopathy, AKI.docxSimulation Case - Status Epilepticus.docxEvaluation Form.docx [file mep_2374-8265.10931-s001.zip › D. Evaluation Form.docx]

We are very interested in your experience and opinions regarding today’s simulation and debriefing. Your feedback is anonymous; your input will be used to improve our efforts.

| **CASE:** | **Strongly Agree** | **Agree** | **Neutral** | **Disagree** | **Strongly Disagree** |
| --- | --- | --- | --- | --- | --- |
| The case content was relevant to my clinical practice. |  |  |  |  |  |
| The case content was sufficiently challenging. |  |  |  |  |  |
| The number of learners was appropriate for the case. |  |  |  |  |  |
| In the debriefing, I felt the learners had the opportunity to speak and were listened to. |  |  |  |  |  |
| In the debriefing, both medical and teamwork/communication issues were addressed. |  |  |  |  |  |
|  |  |  |  |  |  |
| Today’s event will improve my clinical practice. |  |  |  |  |  |
|  |  |  |  |  |  |

| What is your provider type?  (Circle One) | Medical Student Resident Fellow RN APN RT Paramedic PA Pharmacist Other: |
| --- | --- |

**Comments (What can we do better, what works well, suggestions):**

**Name of faculty member(s) leading Sim:**

**(1)__________________________ (2)__________________________ (3)_______________________**

**Rate the teaching effectiveness of the person(s) named above (circle one)**

(1)Excellent / Good / Fair / Poor (2) Excellent / Good / Fair / Poor (3) Excellent / Good / Fair / Poor
